# Acute Effects of Multi-Joint Eccentric Exercise on Lower-Extremity Muscle Activation Measured During Land and Water Walking

**DOI:** 10.3390/muscles4040061

**Published:** 2025-12-10

**Authors:** Brayden Worley, Brennan J. Thompson, Jon Carey, Talin Louder

**Affiliations:** 1 Department of Kinesiology and Health Science, Utah State University, Logan, UT 84322, USA; brayden.worley@usu.edu (B.W.); brennan.thompson@usu.edu (B.J.T.); jon.carey@usu.edu (J.C.); 2 Dennis G. Dolny Movement Research Clinic, Sorenson Legacy Foundation Center for Clinical Excellence, Utah State University, Logan, UT 84322, USA

**Keywords:** electromyography, gait mechanics, co-activation, aquatic therapy, fall prevention

## Abstract

Falls are a leading cause of injury and loss of independence in older adults, often linked to deficits in lower-limb muscle function and gait mechanics. Eccentric exercise can improve muscular resilience, while aquatic walking offers a safe, supportive environment to retrain gait; however, little is known about how these modalities interact at the neuromuscular level. This study compared lower-limb muscle activation during gait on land and in water, before and after an acute bout of eccentric exercise, in healthy young adults. Surface electromyography was collected from the tibialis anterior (TA), gastrocnemius medialis (GM), vastus lateralis (VL), and biceps femoris (BF) during treadmill walking on land and at equivalent speeds in chest-deep water. Results showed that aquatic walking consistently altered activation patterns relative to land walking, with increased TA activity (28%, Cohen’s *d* = 0.69) and reduced GM activity (−27%, Cohen’s *d* = −0.48) during swing, reduced VL activity during stance (−20%, Cohen’s *d* = −0.43), increased VL activity during swing (46%, Cohen’s *d* = 0.72), and increased BF activity during stance (51%, Cohen’s *d* = 0.63). These changes produced distinct co-activation patterns between the shank and thigh. Eccentric exercise had limited effects overall but increased thigh co-activation during swing in land walking. Findings suggest that eccentric exercise can be safely combined with aquatic walking and highlight the potential of this multimodal approach for enhancing gait mechanics relevant to fall prevention.

## 1. Introduction

Aquatic environments are widely utilized in high-performance sport as recovery and early rehabilitation modalities due to their unique physical properties and therapeutic potential [1]. Water’s natural buoyancy reduces joint loading and impact forces, while hydrostatic pressure and viscosity provide opportunities for muscular activation and cardiovascular engagement without the mechanical strain of land-based exercise [2]. Moreover, the supportive yet resistive nature of water can facilitate safer movement retraining for individuals with impaired coordination, proprioception, or postural stability, such as those recovering from stroke, traumatic brain injury, or age-related neuromuscular decline [2]. Despite this promise, aquatic-based interventions remain underutilized in neurorehabilitation compared to their widespread use in athletic populations, highlighting the need to better understand how aquatic exercise can be systematically implemented to support motor function and neuromuscular recovery.

A critical step toward broader clinical adoption is clarifying the neuromuscular demands of aquatic movement. Aerobic exercise in water has been shown to elicit an anabolic stimulus rather than interfere with resistance training, suggesting potential for strength gains at reduced joint loads [3]. This makes aquatic exercise particularly appealing for individuals with compromised musculoskeletal tolerance. Yet, the extent to which muscle activation patterns differ between land and aquatic walking remains underexplored. For clinicians, quantifying these differences is essential to tailoring aquatic-based rehabilitation programs for populations with impaired gait mechanics or reduced muscle coordination. Walking in water is a safe and accessible form of aerobic and resistance exercise for many clinical populations, but its specific effects on lower-limb recruitment are not well characterized. Establishing baseline muscle activation patterns in healthy adults during aquatic walking is therefore an important foundation for future clinical application.

Several studies have investigated lower-extremity muscle activation during aquatic versus land-based treadmill gait, but findings have been inconsistent [4,5,6,7,8,9]. Methodological concerns, particularly around the reliability of surface electromyography (EMG) during immersion, may explain some variability. However, Silvers and Dolny [10] demonstrated that maximum voluntary contraction (MVC) values were not significantly different between water and land, supporting the validity of aquatic EMG. Earlier studies often relied on cumbersome manual waterproofing methods that introduced experimental variability, but recent advancements in submersion-ready EMG systems have greatly improved data quality and consistency.

Using such technology, Long et al. [11] reported that tibialis anterior (TA) activation during the swing phase was significantly greater in water, while gastrocnemius medialis (GA) activation during stance was reduced. These findings are particularly relevant given the TA’s essential role in dorsiflexion and toe clearance: functions often compromised in individuals with neuromuscular disorders. Such adaptations may translate to clinical benefit, as enhancing TA recruitment and reducing GA dominance could improve dorsiflexor control and mitigate fall risk among older adults and patients with conditions such as peripheral neuropathy or Parkinson’s disease. By simultaneously enhancing TA recruitment and reducing GA dominance, aquatic walking may provide a uniquely favorable environment for retraining dorsiflexor function, improving gait mechanics, and reducing fall risk.

While aquatic exercise offers advantages for safe, low-load strengthening, combining it with eccentric exercise may provide additional therapeutic benefit. Eccentric training involves slow lengthening contractions under load, producing high muscular stress that specifically enhances lower-extremity extensor strength, power, and stiffness [12,13,14]. This is clinically relevant because extensors, such as the quadriceps and plantar flexors, are critically engaged when responding to a loss of balance. During a fall, these muscles act eccentrically to decelerate the downward velocity of the body’s center of mass, providing a last line of defense against uncontrolled collapse. Strengthening extensors through eccentric overload may therefore improve the capacity to recover balance and reduce fall risk. Devices such as the Eccentron (BTE Technologies Inc., Hanover, MD, USA) enable multi-joint eccentric overload training and provide an effective means of targeting these functions.

Eccentric exercise may also induce acute neuromuscular responses that influence subsequent walking. For example, fatigue of agonist muscles can shift recruitment toward synergists or antagonists [15], while eccentric contractions have been linked to modifications in spinal reflex excitability [15]. These mechanisms may alter gait dynamics in ways that complement the neuromuscular demands of aquatic walking, though this relationship has not been well studied.

To date, no study has compared lower-extremity muscle activity during aquatic and land-based walking before and after a bout of multi-joint eccentric exercise. Addressing this gap may reveal mechanisms by which a multimodal approach, combining eccentric and aquatic training, optimizes the balance between flexor and extensor activation, enhances muscular strength and stiffness, and ultimately reduces fall risk. Given the prevalence of impaired gait and postural control among older adults and individuals with neuromuscular or orthopedic conditions, these findings may have meaningful translational potential for clinical rehabilitation. Therefore, the purpose of this study was to analyze lower-extremity muscle activation in young adults walking in aquatic and land environments before and after eccentric exercise. Findings from this work may provide preliminary evidence to support future clinical applications of multimodal exercise interventions aimed at improving gait and reducing falls in vulnerable populations.

## 2. Results

### 2.1. Inter-Trial Reliability

Inter-trial reliability of the measured variables ranged from moderate to excellent, except for stance phase co-activation (Co-A) Shank during pre-eccentric land walking, stance phase Co-A Shank during water walking, and swing phase Co-A Shank during pre-eccentric water walking. These exceptions demonstrated lower consistency across trials (*F* = 4.7–583.0, *p* < 0.001; Table 1, Table 2, Table 3 and Table 4).

### 2.2. Repeated Measures Analysis of Variance

#### 2.2.1. Interactions

Measures of central tendency and dispersion for all dependent variables are presented in Table 5. A significant environment × eccentric exercise interaction was observed for Thigh Co-A during the swing phase (*F* = 5.4, *p* < 0.001). Post hoc comparisons revealed that muscle activation during swing was significantly greater following eccentric exercise during land walking compared to pre-eccentric land walking (*p* = 0.005). Additionally, Thigh Co-A during swing was significantly lower during both pre- and post-eccentric water walking compared to post-eccentric land walking (*p* < 0.001). Thigh Co-A was also significantly reduced in pre-eccentric water walking compared to pre-eccentric land walking (*p* = 0.014). The environment × eccentric exercise interaction for vastus lateralis (VL) root-mean-square muscle activation (RMS) during swing approached significance (*F* = 3.4, *p* = 0.067). No other significant interaction effects were observed across remaining variables (*F* = 0.1–0.9, *p* = 0.245–0.822).

#### 2.2.2. Main Effects

Main effects of environment were observed across nearly all dependent measures (*F* = 4.5–602.6, *p* < 0.001–0.036; Table 6), except for biceps femoris (BF) RMS during the swing phase (*F* = 0.2, *p* = 0.699). Several variables increased as a result of water immersion (Table 6), including stance time (*F* = 35.3, *p* < 0.001), swing time (*F* = 602.6, *p* < 0.001), stride length (*F* = 314.9, *p* < 0.001), BF RMS during stance (*F* = 10.2, *p* = 0.002), TA RMS during swing (*F* = 12.2, *p* < 0.001), VL RMS during swing (*F* = 12.9, *p* < 0.001), and Co-A Shank during stance (*F* = 10.8, *p* = 0.001).

In contrast, stride rate (*F* = 406.6, *p* < 0.001), TA RMS during stance (*F* = 6.8, *p* = 0.011), GM RMS during stance (*F* = 26.1, *p* < 0.001), VL RMS during stance (*F* = 4.5, *p* = 0.036), GM RMS during swing (*F* = 5.7, *p* = 0.019), Co-A Thigh during stance (*F* = 4.6, *p* = 0.034), Co-A Shank during swing (*F* = 5.9, *p* = 0.017), and Co-A Thigh during swing (*F* = 35.8, *p* < 0.001) all significantly decreased during water immersion.

A significant main effect of eccentric exercise was observed for Co-A Thigh during the swing phase (*F* = 7.4, *p* = 0.008), with greater muscle co-activation following eccentric exercise (Table 7). No other main effects of eccentric exercise were observed across the remaining dependent variables (*F* = 0.0–2.1, *p* = 0.154–0.929).

## 3. Discussion

The purpose of this study was to compare lower-limb muscle activation during gait in water and on land, before and after a short bout of eccentric exercise, to provide preliminary insight into how a multimodal approach may influence gait mechanics relevant to fall risk. Because the sample consisted of healthy young adults, these results should be interpreted cautiously and viewed as foundational evidence rather than direct indicators of clinical efficacy. Furthermore, the magnitude of observed changes was generally small, and their functional significance remains to be determined. The main findings were that (1) aquatic walking elicited modest and consistent alterations in muscle activation patterns relative to land walking, particularly favoring TA activity and reducing GM activity; (2) thigh muscle responses to water immersion diverged from the shank, showing both reduced VL activity during stance and increased BF activity; (3) eccentric exercise did not broadly disrupt gait mechanics, though a significant environment × eccentric interaction indicated increased thigh co-activation during swing in land walking only. Collectively, these findings suggest that combining eccentric loading with aquatic walking can slightly modulate lower-limb neuromuscular recruitment without impairing stride kinematics; however, these effects should be considered preliminary and specific to young, healthy adults. Future research involving older adults or individuals with neuromuscular impairments is warranted to evaluate the translational potential of these adaptations.

### 3.1. Shank Muscle Activation and Co-Activation

The changes in TA and GM activation observed between water and land align with prior aquatic gait literature [11]. Increased TA activation during swing (+28%, Cohen’s *d* = 0.69) and decreased GM activation during swing (–27%, Cohen’s *d* = −0.48) contributed to a 35% reduction (Cohen’s *d* = −0.49) in shank co-activation during this phase, likely reflecting the reduced need for plantar flexor control and the increased dorsiflexor demand for toe clearance in water. Conversely, during stance, both TA and GM activation decreased, but the relative reduction in GM (–34%, Cohen’s *d* = −1.03) was larger in contrast with the TA (+16%, Cohen’s *d* = −0.51), resulting in greater shank co-activation (+33%, Cohen’s *d* = 0.86). This suggests that water immersion shifts the balance of control toward dorsiflexors during swing and away from plantar flexors during stance, though the magnitude of these effects is moderate and should be interpreted cautiously. These mechanisms could have clinical relevance for individuals with dorsiflexor weakness or impaired toe clearance; however, such implications remain speculative until verified in clinical populations.

### 3.2. Thigh Muscle Activation

While shank findings were anticipated, thigh muscle results revealed a more complex pattern. VL activity was reduced during stance (−20%, Cohen’s *d* = −0.43) and increased during swing (+46%, Cohen’s *d* = 0.72) in water, consistent with buoyancy decreasing extensor demand in stance and viscosity increasing resistance to knee extension in swing. In contrast, BF activity increased during stance (+51%, Cohen’s *d* = 0.63), leading to reduced thigh co-activation (−24%, Cohen’s *d* = −0.43). These results suggest that water immersion reduces reliance on quadriceps extensors while enhancing hamstring recruitment, possibly due to buoyancy unloading vertical forces while fluid resistance challenges posterior chain control. Although these adaptations were observable, the changes were relatively small, and their functional impact on gait remains uncertain. Further investigation is necessary to determine whether similar neuromuscular responses occur in clinical or aging populations. This inversion between shank and thigh co-activation patterns underscores the environment-specific redistribution of neuromuscular demand during aquatic walking, but conclusions regarding functional benefits should remain tentative.

### 3.3. Post-Eccentric Thigh Co-Activation

Contrary to expectations, eccentric exercise did not broadly alter TA or GM activity across environments. This may reflect the loading profile of the multi-joint isokinetic dynamometer, which emphasizes quadriceps rather than plantar flexor eccentric stress [16]. The single significant interaction, greater thigh co-activation during swing following eccentric exercise in land walking, may be partially explained by a reduction in VL activity post-exercise, consistent with localized quadriceps fatigue. While this effect is modest, it highlights the sensitivity of quadriceps-dominant tasks to eccentric overload and warrants further study on how eccentric fatigue interacts with gait demands in different environments. Overall, the current findings represent an initial, preliminary step toward understanding the compatibility of eccentric and aquatic modalities, with caution advised in extrapolating to clinical populations. Future studies are needed to explore whether these small neuromuscular changes translate into meaningful functional adaptations in populations where balance, strength, or motor control are compromised.

### 3.4. Implications, Limitations, and Future Directions

These findings suggest that eccentric training and aquatic walking can be safely combined, at least in young adults, without disrupting stride mechanics or lower-limb activation patterns. Importantly, extensors such as the quadriceps and plantar flexors act eccentrically to decelerate the body’s center of mass during balance recovery. Enhancing their strength through eccentric exercise, while concurrently retraining gait mechanics in water, may offer a complementary pathway for improving both muscular resilience and dynamic stability. The reduced co-activation demands observed in water may also provide a safer and more accessible training environment for populations with impaired gait.

Several limitations must be acknowledged. First, participants were healthy, recreationally active young adults, which limits generalizability to older adults or clinical populations who may benefit most from fall-prevention strategies. Second, the use of body mass index (BMI) as an inclusion criterion and as a factor influencing EMG signal quality represents a limitation, as BMI may not adequately account for the effects of subcutaneous fat on EMG signal integrity. Incorporating direct body composition assessments in future studies could improve accuracy and generalizability. Finally, transitioning between aquatic and land conditions may have introduced minor electrode movement artifacts that could have affected EMG consistency.

Future studies should test longitudinal multimodal programs in older adults or those with gait impairments, using population-specific eccentric loading parameters and aquatic walking protocols (e.g., water depth, speed, jet resistance). In doing so, researchers should incorporate direct body composition assessments to address the limitations associated with relying on BMI, an issue noted earlier, as BMI alone may insufficiently capture the influence of subcutaneous fat on EMG signal quality. Such work is needed to determine whether the small neuromuscular changes observed here translate into meaningful functional adaptations and potential reductions in fall risk.

### 3.5. Conclusions

This study demonstrates that aquatic walking alters lower-limb muscle activation in systematic ways, favoring dorsiflexor recruitment in the shank and shifting thigh demand toward the hamstrings. Eccentric exercise produced limited acute effects but did not disrupt gait mechanics, supporting its compatibility with aquatic training. These preliminary findings support the feasibility of integrating eccentric and aquatic exercise as a multimodal approach for enhancing lower-extremity function and potentially reducing fall risk in at-risk populations.

## 4. Materials and Methods

### 4.1. Participants

An a priori power analysis was conducted using G*Power (version 3.1.9.7) [17], based on a medium effect size (*f* = 0.3) [11], an alpha level of 0.05, and a desired power of 0.80. The analysis indicated that a minimum sample size of 24 participants would be required to achieve adequate statistical power. To meet this requirement, we recruited a convenience sample of 26 healthy, recreationally active adults (see Table 8. All participants successfully completed the full study protocol.

Eligibility criteria included being between the ages of 18 and 35 and self-reporting that they engaged in regular physical activity. Participants were excluded if they: (a) reported a history of neurological conditions associated with motor symptoms (e.g., stroke, multiple sclerosis, recent concussion); (b) experienced current pain or injury affecting gait; (c) underwent surgical intervention to the lower limbs or trunk within the past two years; or (d) sustained a ligament tear in the hip, knee, or ankle within the same time frame.

All participants provided written informed consent, as approved by the University’s Institutional Review Board.

### 4.2. Procedures

**Figure 1:**
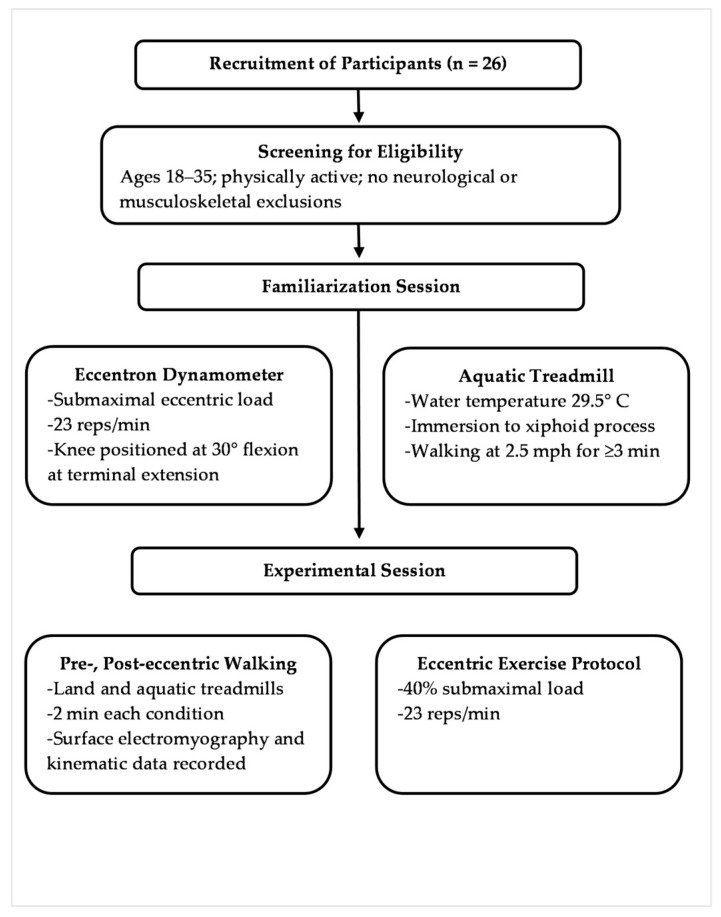
Study design flow chart.

Refer to Figure 1 for a detailed flow chart of study procedures. Participants completed a dedicated familiarization session prior to experimental testing to acclimate to both the multi-joint eccentric dynamometer (Eccentron; BTE Technologies, Hanover, MD, USA) and the aquatic treadmill (HydroWorx 2000; HydroWorx, Middletown, PA, USA). Both the familiarization and experimental sessions were conducted in the same Motion Analysis Laboratory to ensure consistency in testing environment and equipment setup.

The familiarization session began with eccentric exercise on the Eccentron dynamometer at a submaximal load of 40%, selected to simulate the demands of the subsequent experimental protocol. This load was determined individually for each participant based on the results of a maximal eccentric strength assessment conducted at the start of the session. A movement cadence of 23 repetitions per minute was selected for the dynamometer, consistent with previous protocols [18,19]. The seat was adjusted to position the knee at 30° of flexion at terminal extension, ensuring standardized joint alignment across participants.

Following Eccentron familiarization, participants were introduced to the aquatic treadmill. Water temperature was maintained at 29.5 ± 0.2 °C during both the familiarization and experimental sessions, aligning with the thermoneutral range recommended for aquatic exercise [20]. During all instances of aquatic walking, participants were submerged to the level of the xiphoid process. This immersion depth has been shown to reduce buoyancy-related float time during the swing phase of gait, promoting more natural stride mechanics [21]. They were instructed to walk at a speed of 2.5 mph for a minimum of 3 min. Familiarization was considered successful when participants demonstrated a consistent, “normal” gait pattern, operationally defined as underwater walking that closely resembled their typical over ground gait. This was confirmed through visual inspection using the aquatic treadmill’s built-in underwater cameras. Specific criteria for normal gait included: (1) a rhythmic and reciprocal stepping pattern; (2) the absence of prolonged float or flight phases between steps; (3) proper foot placement with heel or mid-foot contact followed by toe-off; (4) coordinated arm swing that did not involve propulsive or swimming-like movements; (5) trunk alignment consistent with upright walking posture. Anthropometric data, including height, weight, and body mass index (BMI), were also collected during this session.

Experimental testing for each participant was completed in a single session, scheduled at least 72 h after the familiarization session to minimize residual effects. At the start of the session, electrode preparation involved shaving and cleansing the skin at each electrode site with alcohol swabs. Waterproof, adhesive electrodes were then placed over the TA, GM, BF, and VL muscles of the dominant leg, in accordance with the guidelines established by the Surface Electromyography for the Non-Invasive Assessment of Muscles (SENIAM) project [22]. To further minimize motion artifact and enhance signal fidelity, electrodes were secured with hypoallergenic, waterproof adhesive wrap. Leg dominance was operationally defined as the leg the participant would use to kick a ball.

Experimental testing involved four 2 min walking trials at 2.5 mph, comprising both land treadmill (Tandem Treadmill, AMTI, Watertown, MA, USA) and water treadmill conditions, conducted before and after a 3 min bout of eccentric exercise. The eccentric protocol was performed at 40% of the participant’s maximal load, with a cadence of 23 repetitions per leg per minute. EMG data were continuously recorded during all walking trials using a 16-channel waterproof EMG system (Cometa Mini Wave; Cometa SRL, Milan, Italy) at a sampling rate of 2000 Hz, as recommended for dynamic gait analysis [4]. Two-dimensional video recordings of participants’ legs were captured from a sagittal-plane perspective at 100 Hz using a waterproof camera (Miqus Underwater; Qualisys AB, Gothenburg, Sweden) synchronized with EMG data. During aquatic trials, participants wore either swimsuits or compression garments and walked barefoot; for land trials, standard athletic footwear was worn.

### 4.3. Data Analysis

Ten full stride cycles from the final 30 s of each walking trial were selected for analysis. A full stride cycle was defined as the time interval between two consecutive foot strikes of the dominant foot. Phase demarcations for stance and swing were identified using frame-by-frame video analysis, with stance defined as the period from foot strike to toe-off, and swing as the period from toe-off to the subsequent foot strike.

EMG signals were processed using MATLAB (version R2023a, The MathWorks Inc., Natick, MA, USA). All 12 sEMG signals were filtered using a 4th-order recursive Butterworth filter with a bandwidth of 10–500 Hz to minimize low-frequency drift and high-frequency noise. Muscle activation magnitudes during the stance and swing phases were estimated by calculating the RMS of the filtered sEMG signals. RMS values were averaged across 10 stride cycles for each phase and condition, and the resulting mean values were used for statistical analysis.

Muscle Co-A indices for both stance and swing phases were computed as linear ratios of antagonist to agonist RMS activation. Two separate Co-A indices were calculated: one for the shank (lower-leg muscles) and one for the thigh. During the swing phase, the TA and VL were classified as agonists, while the GM and BF were considered antagonists. These roles were reversed during the stance phase, with GM and BF as agonists and TA and VL as antagonists.

Additionally, kinematic variables including stance time, swing time, stride length, and stride rate were computed and averaged across the same 10 stride cycles for each condition. All mean values for sEMG and kinematic variables were submitted for statistical analysis.

### 4.4. Statistical Analysis

All statistical analyses were conducted using RStudio (Version 4.3.3). Intraclass correlation coefficients (ICCs) with corresponding 95% confidence intervals (CIs) were calculated to assess the inter-trial reliability of sEMG measurements. ICCs were derived using a single-measure, absolute agreement, two-way mixed-effects model.

To evaluate the effects of environment and eccentric exercise, a two-way repeated-measures analysis of variance (RMANOVA) was conducted for each dependent variable. The within-subject factors included environment (land × water) and eccentric exercise condition (pre-eccentric × post-eccentric). All hypothesis tests were evaluated using a Type I error rate (*α*) of 0.05. ICC values were classified as excellent reliability (>0.90), good reliability (0.75–0.90), moderate reliability (0.50–0.75), or poor reliability (<0.50) [23]. ICC values were interpreted based on established benchmarks, with values > 0.90 indicating excellent reliability, 0.75–0.90 as good reliability, 0.50–0.75 as moderate reliability, and <0.50 as poor reliability, in accordance with the guidelines proposed by Koo and Li [23].

## Figures and Tables

**Table 1:** Inter-trial reliability of land measures during the stance phase.

Measure	Pre-Eccentric	Post-Eccentric
Stance time (s)	0.851 (0.770–0.918)	0.812 (0.714–0.896)
TA RMS (μV)	0.681 (0.548–0.812)	0.716 (0.589–0.835)
GM RMS (μV)	0.843 (0.756–0.914)	0.862 (0.784–0.926)
VL RMS (μV)	0.664 (0.528–0.800)	0.980 (0.966–0.990)
BF RMS (μV)	0.944 (0.907–0.971)	0.979 (0.965–0.989)
Co-A Shank (%)	0.261 (0.143–0.442)	0.631 (0.490–0.776)
Co-A Thigh (%)	0.631 (0.490–0.776)	0.631 (0.490–0.777)

Data are presented as ICC estimate (95% confidence interval). TA = tibialis anterior; GM = medial gastrocnemius; VL = vastus lateralis; BF = biceps femoris; RMS = root-mean-square; Co-A = co-activation. Surface electromyography data were collected from 26 healthy, recreationally active young adults during 2 min walking trials (2.5 mph) on both a land-based and underwater treadmill, completed before and after a 3 min bout of submaximal (40%) eccentric exercise performed on a seated, multi-joint lower-extremity eccentric dynamometer.

**Table 2:** Inter-trial reliability of water measures during the stance phase.

Measure	Pre-Eccentric	Post-Eccentric
Stance time (s)	0.794 (0.690–0.885)	0.772 (0.660–0.872)
TA RMS (μV)	0.683 (0.550–0.813)	0.725 (0.601–0.841)
GM RMS (μV)	0.585 (0.440–0.742)	0.539 (0.392–0.706)
VL RMS (μV)	0.740 (0.620–0.851)	0.983 (0.972–0.991)
BF RMS (μV)	0.781 (0.672–0.877)	0.802 (0.700–0.890)
Co-A Shank (%)	0.421 (0.278–0.605)	0.422 (0.279–0.606)
Co-A Thigh (%)	0.571 (0.425–0.731)	0.558 (0.411–0.721)

Data are presented as ICC estimate (95% confidence interval). TA = tibialis anterior; GM = medial gastrocnemius; VL = vastus lateralis; BF = biceps femoris; RMS = root-mean-square; Co-A = co-activation. Surface electromyography data were collected from 26 healthy, recreationally active young adults during 2 min walking trials (2.5 mph) on both a land-based and underwater treadmill, completed before and after a 3 min bout of submaximal (40%) eccentric exercise performed on a seated, multi-joint lower-extremity eccentric dynamometer.

**Table 3:** Inter-trial reliability of land measures during the swing phase.

Measure	Pre-Eccentric	Post-Eccentric
Stance time (s)	0.763 (0.651–0.864)	0.787 (0.680–0.881)
TA RMS (μV)	0.725 (0.600–0.841)	0.915 (0.862–0.955)
GM RMS (μV)	0.627 (0.486–0.774)	0.923 (0.875–0.960)
VL RMS (μV)	0.678 (0.544–0.810)	0.964 (0.940–0.981)
BF RMS (μV)	0.878 (0.807–0.935)	0.970 (0.950–0.985)
Co-A Shank (%)	0.609 (0.466–0.761)	0.776 (0.666–0.874)
Co-A Thigh (%)	0.769 (0.657–0.870)	0.746 (0.627–0.855)

Data are presented as ICC estimate (95% confidence interval). TA = tibialis anterior; GM = medial gastrocnemius; VL = vastus lateralis; BF = biceps femoris; RMS = root-mean-square; Co-A = co-activation. Surface electromyography data were collected from 26 healthy, recreationally active young adults during 2 min walking trials (2.5 mph) on both a land-based and underwater treadmill, completed before and after a 3 min bout of submaximal (40%) eccentric exercise performed on a seated, multi-joint lower-extremity eccentric dynamometer.

**Table 4:** Inter-trial reliability of water measures during the swing phase.

Measure	Pre-Eccentric	Post-Eccentric
Stance time (s)	0.868 (0.792–0.929)	0.812 (0.714–0.896)
TA RMS (μV)	0.846 (0.760–0.916)	0.886 (0.819–0.939)
GM RMS (μV)	0.503 (0.356–0.677)	0.525 (0.378–0.695)
VL RMS (μV)	0.539 (0.392–0.706)	0.987 (0.977–0.993)
BF RMS (μV)	0.692 (0.561–0.819)	0.934 (0.891–0.965)
Co-A Shank (%)	0.487 (0.341–0.663)	0.703 (0.574–0.827)
Co-A Thigh (%)	0.580 (0.434–0.739)	0.836 (0.747–0.910)

Data are presented as ICC estimate (95% confidence interval). TA = tibialis anterior; GM = medial gastrocnemius; VL = vastus lateralis; BF = biceps femoris; RMS = root-mean-square; Co-A = co-activation. Surface electromyography data were collected from 26 healthy, recreationally active young adults during 2 min walking trials (2.5 mph) on both a land-based and underwater treadmill, completed before and after a 3 min bout of submaximal (40%) eccentric exercise performed on a seated, multi-joint lower-extremity eccentric dynamometer.

**Table 5:** Central tendency and dispersion results.

Measure	Land Pre-Ecc	Land Post-Ecc	Water Pre-Ecc	Water Post-Ecc
Stance Time (s)	0.75 (0.04)	0.73 (0.04)	0.80 (0.06)	0.80 (0.07)
Swing Time (s)	0.40 (0.03)	0.41 (0.03)	0.66 (0.08)	0.69 (0.07)
Stride Length (m)	1.28 (0.06)	1.28 (0.06)	1.63 (0.14)	1.67 (0.14)
Stride Rate (strides*s^−1^)	0.87 (0.04)	0.88 (0.04)	0.69 (0.05)	0.67 (0.06)
TA RMS Stance (μV)	62.4 (21.8)	64.7 (22.0)	51.9 (18.0)	51.6 (20.1)
GM RMS Stance (μV)	85.9 (30.3)	83.6 (37.1)	55.3 (22.1)	56.3 (23.7)
VL RMS Stance (μV)	22.3 (12.4)	22.3 (12.4)	17.6 (7.8)	16.1 (7.2)
BF RMS Stance (μV)	26.4 (24.3)	23.6 (12.8)	36.4 (20.3)	39.0 (21.9)
TA RMS Swing (μV)	78.9 (22.8)	71.3 (33.5)	93.0 (26.8)	99.5 (38.1)
GM RMS Swing (μV)	15.6 (12.1)	14.6 (8.3)	11.0 (5.5)	11.0 (7.9)
VL RMS Swing (μV)	15.9 (11.7)	10.4 (7.7)	18.7 (5.8)	19.4 (7.3)
BF RMS Swing (μV)	34.8 (21.6)	33.4 (18.8)	37.8 (24.5)	33.6 (18.8)
Co-A Shank Stance (%)	74.9 (31.2)	86.3 (41.6)	108.0 (42.3)	106.9 (49.5)
Co-A Thigh Stance (%)	94.6 (41.2)	91.0 (53.7)	66.6 (45.1)	75.3 (63.9)
Co-A Shank Swing (%)	19.3 (14.2)	22.9 (18.7)	14.7 (13.0)	13.0 (14.2)
Co-A Thigh Swing (%)	278.8 (134.1)	426.9 (234.3) ^a^	170.9 (74.8) ^a,b^	182.7 (107.8) ^b^

Pre-Ecc = pre-eccentric; Post-Ecc = post-eccentric; TA = tibialis anterior; GM = medial gastrocnemius; VL = vastus lateralis; BF = biceps femoris; RMS = root-mean-square; Co-A = co-activation. ^a^ Significantly different from Land Pre-Ecc (*p* < 0.05). ^b^ Significantly different from Land Post (*p* < 0.05). Surface electromyography data were collected from 26 healthy, recreationally active young adults during 2 min walking trials (2.5 mph) on both a land-based and underwater treadmill, completed before and after a 3 min bout of submaximal (40%) eccentric exercise performed on a seated, multi-joint lower-extremity eccentric dynamometer.

**Table 6:** Central tendency and dispersion results collapsed across pre- and post-eccentric exercise.

Measure	Land	Water
Stance Time (s)	0.74 (0.04)	0.80 (0.06) ^a^
Swing Time (s)	0.41 (0.03)	0.68 (0.07) ^a^
Stride Length (m)	1.28 (0.06)	1.65 (0.14) ^a^
Stride Rate (strides*s^−1^)	0.87 (0.04)	0.68 (0.06) ^a^
TA RMS Stance (μV)	61.8 (20.1)	51.8 (18.9) ^a^
GM RMS Stance (μV)	84.8 (33.6)	55.8 (22.7) ^a^
VL RMS Stance (μV)	21.1 (12.0)	16.9 (7.5) ^a^
BF RMS Stance (μV)	25.0 (19.3)	37.7 (20.9) ^a^
TA RMS Swing (μV)	75.1 (28.6)	96.3 (32.8) ^a^
GM RMS Swing (μV)	15.1 (10.3)	11.0 (6.8) ^a^
VL RMS Swing (μV)	13.1 (10.2)	19.1 (6.5) ^a^
BF RMS Swing (μV)	34.1 (20.0)	35.7 (21.7)
Co-A Shank Stance (%)	80.6 (36.9)	107.4 (25.6) ^a^
Co-A Thigh Stance (%)	92.8 (47.4)	70.9 (54.9) ^a^
Co-A Shank Swing (%)	21.1 (16.5)	13.8 (13.5) ^a^
Co-A Thigh Swing (%)	353.9 (203.3)	176.8 (92.1) ^a^

TA = tibialis anterior; GM = medial gastrocnemius; VL = vastus lateralis; BF = biceps femoris; RMS = root-mean-square; Co-A = co-activation. ^a^ Significantly different from Land (*p* < 0.05). Surface electromyography data were collected from 26 healthy, recreationally active young adults during 2 min walking trials (2.5 mph) on both a land-based and underwater treadmill, completed before and after a 3 min bout of submaximal (40%) eccentric exercise performed on a seated, multi-joint lower-extremity eccentric dynamometer.

**Table 7:** Central tendency and dispersion results collapsed across environment.

Measure	Pre-Eccentric	Post-Eccentric
Stance Time (s)	0.77 (0.06)	0.77 (0.06)
Swing Time (s)	0.53 (0.14)	0.55 (0.15)
Stride Length (m)	1.46 (0.21)	1.47 (0.22)
Stride Rate (strides*s^−1^)	0.78 (0.10)	0.78 (0.11)
TA RMS Stance (μV)	55.4 (18.1)	58.2 (21.9)
GM RMS Stance (μV)	70.6 (30.5)	69.9 (33.7)
VL RMS Stance (μV)	19.9 (10.5)	18.0 (9.8)
BF RMS Stance (μV)	31.4 (22.7)	31.3 (19.4)
TA RMS Swing (μV)	86.0 (25.6)	85.4 (38.3)
GM RMS Swing (μV)	13.3 (9.6)	12.8 (8.3)
VL RMS Swing (μV)	17.3 (9.3)	14.9 (8.7)
BF RMS Swing (μV)	36.3 (22.9)	33.5 (18.6)
Co-A Shank Stance (%)	91.4 (40.4)	96.6 (46.5)
Co-A Thigh Stance (%)	80.6 (45.1)	83.1 (59.0)
Co-A Shank Swing (%)	17.0 (13.7)	17.9 (17.2)
Co-A Thigh Swing (%)	224.9 (120.6)	304.8 (218.7) ^a^

TA = tibialis anterior; GM = medial gastrocnemius; VL = vastus lateralis; BF = biceps femoris; RMS = root-mean-square; Co-A = co-activation. ^a^ Significantly different from Pre-Eccentric (*p* < 0.05). Surface electromyography data were collected from 26 healthy, recreationally active young adults during 2 min walking trials (2.5 mph) on both a land-based and underwater treadmill, completed before and after a 3 min bout of submaximal (40%) eccentric exercise performed on a seated, multi-joint lower-extremity eccentric dynamometer.

**Table 8:** Participant characteristics.

Sex	n	Age (years)	Height (cm)	Body Mass (kg)	BMI
Total	26	21.6 (2.5)	176.4 (6.9)	75.9 (13.3)	24.2 (3.4)
Female	10	20.3 (2.2)	174.9 (4.4)	76.1 (10.9)	24.9 (3.3)
Male	16	22.4 (2.4)	177.3 (8.2)	75.7 (14.9)	24.1 (3.5)

Data are reported as mean (SD); BMI = body mass index.

## Data Availability

The raw data supporting the conclusion of this article will be made available by the authors upon request.
